# Association of the *CCR5*Δ32 variant with juvenile idiopathic arthritis in a meta-analysis

**DOI:** 10.1186/1546-0096-9-S1-P287

**Published:** 2011-09-14

**Authors:** THCM Reinards, HM Albers, DMC Brinkman, SSM Kamphuis, MAJ van Rossum, EPAH Hoppenreijs, HJ Girschick, C Wouters, RK Saurenmann, JJ Houwing-Duistermaat, REM Toes, TWJ Huizinga, R ten Cate, MW Schilham

**Affiliations:** 1 Leiden University Medical Center, Leiden, The Netherlands; 2 Erasmus Medical Center, Rotterdam, The Netherlands; 3 Academic Medical Center, Amsterdam, The Netherlands; 4 Radboud University Medical Center, Nijmegen, The Netherlands; 5 University of Würzburg, Würzburg, Germany; 6 University Hospital Gasthuisberg, Leuven, Belgium; 7 University Children's Hospital, Zürich, Switzerland

## Background

CCR5 is expressed on Th1 cells and may play a role in Rheumatoid Arthritis by recruiting these cells to the synovium, where they drive an inflammatory process. The *CCR5*Δ32 variant, a deletion variant which leads to a dysfunctional receptor, has been reported in several genetic association studies in Juvenile Idiopathic Arthritis (JIA), with conflicting results. CCL14 is one of the ligands of CCR5 and polymorphisms in the *CCL14* gene have been reported to be associated with Systemic Lupus Erythematosus.

## Aim

We performed a case-control genetic association study to investigate whether *CCR5* and *CCL14* polymorphisms are associated with susceptibility to JIA.

## Methods

*CCR5*Δ32 and *CCL14* rs16971802 were genotyped in 667 JIA cases and 1320 healthy controls, both of North-West-European white origin. Patients with oligoarticular (persistent and extended), polyarticular (rheumatoid factor negative and positive) and systemic JIA have been included. A meta-analysis combined with three published studies on *CCR5*Δ32 in JIA, with comparable allele frequencies in controls, was performed.

## Results

*CCR5*Δ32 and *CCL14* rs16971802 were not significantly associated with JIA in this study, with p-values of 0.12 and 0.72 respectively. Nevertheless, meta-analysis demonstrated association of *CCR5*Δ32 with protection to JIA (combined p=0.0003, OR=0.83, 95% CI: 0.75-0.91, Breslow-Day p=0.87, heterogeneity I-squared=0.0%).

## Conclusion

This study has not demonstrated significant associations of *CCR5* and *CCL14* polymorphisms with JIA, but the association of *CCR5*Δ32 with protection from developing JIA is strengthened in a meta-analysis. It is hypothesized that function of CCR5 could influence synovial inflammation also in JIA.

